# BIOLOGICAL THERAPY OF PSORIASIS

**DOI:** 10.4103/0019-5154.62754

**Published:** 2010

**Authors:** Raja K Sivamani, Genevieve Correa, Yoko Ono, Michael P Bowen, Siba P Raychaudhuri, Emanual Maverakis

**Affiliations:** 1 *From the Department of Medicine, Santa Clara Valley Medical Center, San Jose, CA 95128*; 2 *From the Department of Dermatology, School of Medicine, University of California, Davis, Sacramento, CA 95817*; 3 *From the Veterans Affairs Northern California Health Care System, Sacramento, CA 95655*; 4 *From the Division of Rheumatology, Allergy and Clinical Immunology, University of California, Davis, Sacramento, CA 95817.*

**Keywords:** *Adverse effects*, *biologics*, *psoriasis*, *therapy*

## Abstract

The treatment of psoriasis has undergone a revolution with the advent of biologic therapies, including infliximab, etanercept, adalimumab, efalizumab, and alefacept. These medications are designed to target specific components of the immune system and are a major technological advancement over traditional immunosuppressive medications. These usually being well tolerated are being found useful in a growing number of immune-mediated diseases, psoriasis being just one example. The newest biologic, ustekinumab, is directed against the p40 subunit of the IL-12 and IL-23 cytokines. It has provided a new avenue of therapy for an array of T-cell-mediated diseases. Biologics are generally safe; however, there has been concern over the risk of lymphoma with use of these agents. All anti-TNF-α agents have been associated with a variety of serious and “routine” opportunistic infections.

## Introduction

**Figure 1:**
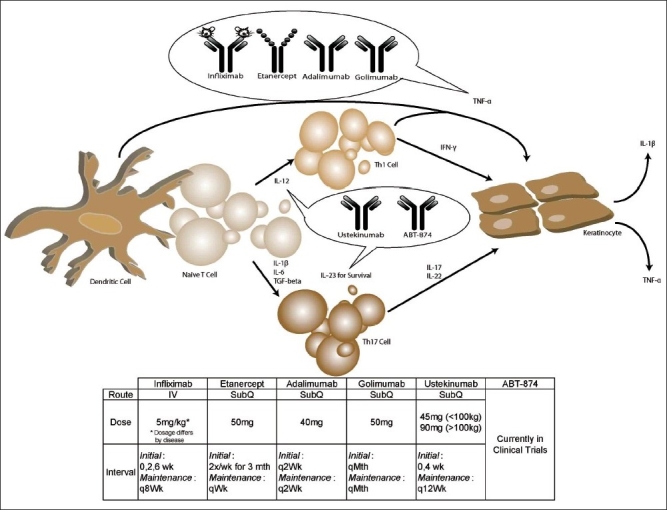
Biologics in psoriasis and their possible mechanisms. TNF- α secreted by antigen-presenting cells; Th-1 cells and keratinocytes can be neutralized by the anti-TNF biologics infliximab, etanercept, adalimumab, and golimumab. Adalimumab and golimumab are fully human antibodies directed against TNF-α. Infliximab was developed from a mouse anti-TNF antibody that was then partially humanized. Etanercept is a molecullarly engineered molecule formed by linking the TNF-α receptor to the Fc portion of an antibody. Ustekinumab and ABT-874 are directed against the p40 subunit of IL-12 and IL-23. IL-12 is needed for differentiation of naive cells into Th-1 cells and IL-23 is needed for the maintenance of IL-17-secreting Th17 cells. IFN-α secreted by Th-1 cells and IL-17 and IL-22 secreted by Th-17 cells activate keratinocytes, which in turn proliferate and secrete IL-12 and TNF-α.

Psoriasis is a life-long chronic inflammatory skin condition affecting approximately 2% of the general population.[1,2] There are many clinical variants of psoriasis. Most patients have plaques with silver-white scale and an erythematous base. Some patients have joint involvement. There is strong evidence in favor of psoriasis being an immune-mediated disease with T-cells playing a central role.[3,4] However, the pathogenesis of psoriasis is complex and likely includes mediators of both the innate and adaptive immune systems. In support of an immune etiology, psoriasis can either develop or go into remission following a bone marrow transplantation.[5,6] To date, there is no consensus as to the antigens involved in the autoreactive immune response that is responsible for psoriasis. However, the cytokine secretion profile of the T-cells has been well characterized and both Th1 and Th17 cells have been found to play a role in the pathogenesis of psoriasis.[7] Th1 differentiation is mediated by IL-12. In contrast, Th17 cells develop in the presence of IL-1, IL-6, and TGF-α. Once differentiated, IL-23 is then required for their maintanance. Th1 cells release mediators such as TNF-α and IFN-α that lead to vasodilation, leukocyte migration and activation of keratinocytes.[4] This in turn leads to further activation of dendritic cells, creating a cycle of inflammation. Th-17 cells also stimulate keratinocyte activation and proliferation through secretion of IL-17 and IL-22.[8–10] A schematic of the activation process is shown in Figure 1.

Biological therapy is the use of agents that can specifically target an immune or genetic mediator of a pathophysiological process. The introduction of biological-based therapies has greatly improved treatment of psoriasis. Several biological therapies have emerged over the past decade for psoriasis alone [Table 1]. Earlier agents disrupted activation and migration of T-cells and these include alefacept and efalizumab. Later agents have targeted TNF-α and these include infliximab, etanercept, and adalimumab. Recently, agents that target the p40 subunit shared by both IL-12 and IL-23 have been developed and these include ustekinumab and ABT-874. The sites of action of the anti-TNF and the anti-IL12/IL23 agents are indicated in Figure 1. Clinical trials that have investigated the role of biologics in psoriasis therapy are reviewed in Table 2a and 2b.

**Table 1:** Biologics in treatment of psoriasis

Biologic	Immunological construct	Mechanism of action	Manufacturer	Route
Alefacept	Human fusion protein of the first extracellular domain of LFA-3 fused Fc portion of human IgG1	LFA-3 portion binds to CD2 on memory T-cells to block their activation. Fc portion binds to CD 16 on natural killer cells to induce apoptosis of memory T-cells	Astellas Pharma USA, Inc.	IV
Infliximab	Chimeric (murine-human) antibody against TNF-α	Binds TNF to neutralize its effects	Centocor Ortho Biotech Inc.	IV
Etanercept	Human fusion protein of the TNF receptor to Fc portion of IgG1	Binds TNF to neutralize its effects	Amgen® and Wyeth®	SC
Adalimumab	Human monoclonal antibody against TNF	Binds TNF to neutralize its effects	Abbot Laboratories	SC
Golimumab	Human monoclonal antibody against TNF	Binds TNF to neutralize its effects	Centocor Ortho Biotech Inc.	SC
Ustekinumab	Human monoclonal antibody against the p40 subunit of IL-12 and IL-23 from human immunoglobulin transgenic mice	Blocks the actions of IL-12 and IL-23	Centocor Ortho Biotech Inc.	SC
ABT-874	Human monoclonal antibody against the p40 subunit of IL-12 and IL-23 isolated from human anti body phage display library	Blocks the actions of IL-12 and IL-23	Abbot Laboratories	SC

**Table 2a:** Clinical trials of biologics for psoriasis

Biologic	Study	Study design	Duration of study	Dosing	Antibody formation against Bologic
Alefacept	Phase 2 – 229 patients with CPP[1] Phase 3 – 553 patients with CPP[2] Phase 3 – 507 patients with CPP[3]	DB, PC, PG study at 22 sites in USA DB, PC, PG study at 51 sites in USA and Canada with crossover at 12 weeks DB, PC, PG study at 64 sites in USA, Canada, and Europe	12 week treatment phase with a 12 week follow-up 12 week treatment phase with a 12 week followup, followed by another 12 week treatment phase with a 12 week follow up; Cohort 1: Alefacept-Alefacept, Cohort 2: Alefacept-Placebo, Cohort 3: Placebo-Alefacept 12 week treatment phase with 12 week follow-up	IV once a week: placebo, 0.025 mg/kg, 0.075 mg/kg, 0.15 mg/kg IV once a week: placebo, 7.5 mg IM once a week: placebo, 10 mg, 15 mg	One patient developed “low” antibody titer Five patients developed “low” antibody titers 4% of patients tested in alefacept-treated patients; Antibodies were not neutralizing and had titers<1:40; one of the placebo patients had antialefacept antibodies
Infliximab	Phase 2 – 249 patients with CPP[4] Phase 3 – 378 patients with CPP Phase 3 – 835 patients with CPP[5]	DB, PC, PG study at 24 sites in USA DB, PC, PG study at 32 sites in Canada and Europe with placebo crossover DB, PC, PG study at 63 sites in USA, Canada, and Europe with rerandomization to scheduled or “as needed” treatment	6 week induction treatment phase with a 20 week follow-up 6 week induction phase with placebo-controlled treatment phase to 24 weeks followed by placebo crossover to 46 weeks 6 week induction phase followed by rerandomization to either “as needed” infusions or scheduled 8 week infusions to 50 weeks	IV given at week 0, 2, and 6: placebo, 3 mg/kg, 5 mg/kg IV given at week 0, 2, and 6 and then every 8 weeks: placebo, 5 mg/kg; at 24 weeks placebo crossed over to receive 5 mg/kg IV given at week 0, 2, and 6: placebo, 3mg/kg, 5 mg/kg. Re-randomized at week 14 to either scheduled or “as needed” dosing. Placebo started scheduled dosing (5 mg/kg) at 8 weeks	27% and 20% of patient in 3 mg/kg and 5 mg/kg, respectively Cumulatively 27% of patients formed antibodies; antibody formation associated with loss of response At week 66, 49% and 39% of patients formed antibodies in the 3 mg/kg and 5 mg/kg treatment groups, respectively; 61.5% of titers were<1:40; antibody formation was related to loss of response
Etanercept	Phase 2 – 112 patients with CPP[6] Phase 3 – 652 patients with CPP[7] Phase 3 – 611 patients with CPP[8] Phase 3 – 618 patients with CPP[9] Phase 3 – 211 children with CPP[10]	DB, PC, PG multiple sites in USA DB, PC, PG at 47 sites in USA with placebo crossover DB, PC, PG at 50 sites in USA, Canada, Western Europe followed by open label treatment phase DB, PC, PG at 39 sites in USA and Canada with open label extension DB, PC, PG at 42 sites in USA and Canada followed by open label and then double-blind withdrawalreadministration phase	24 week treatment phase 12 week placebo-controlled treatment phase followed by another 12 week treatment phase where placebo was crossed over to treatment group 12 week placebo-controlled treatment phase followed by a 12 week open label treatment phase 12 week placebo-controlled treatment phase followed by open label extension to 96 weeks 12 week placebo-controlled treatment phase followed by 24 week open label phase followed by a 12 week double blind withdrawalreadministration phase	Subcutaneous given every other week: placebo, 25 mg Subcutaneous: placebo, low (25 mg/week), medium (25 mg twice a week), high (50 mg twice a week); After 12 weeks placebo received medium dosing Subcutaneous: placebo, 25 mg, 50 mg twice weekly; After 12 weeks all patients received 25 mg twice weekly Subcutaneous: placebo, 50 mg twice weekly; after 12 weeks, all patients received 50 mg twice weekly for total treatment of 96 weeks Subcutaneous: placebo, 0.8 mg/kg (up to 50 mg) per week	Not reported Eight patients developed antibodies and no titers reported 1.1% and 1.6% developed antibodies in first and second treatment phases respectively; antibodies did not affect efficacy; 73% of these patients had no antibodies at subsequent testing 18.3% of patients had antibodies and titers were not reported; presence of antibody did not affect efficacy of treatment Not reported
Adalimumab	Phase 2 – 147 patients with CPP[11] Phase 3 – 271 patients with CPP[12] Phase 3 – 1212 patients with CPP[13]	DB, PC, PG at 18 sites in USA and Canada with placebo crossover and open label extension DB, PC, PG at 28 sites in Europe and Canada DB, PC, PG at 81 sites in USA and Canada with placebo crossover openlabel extension and blinded withdrawal	12 week placebo controlled treatment phase followed by a 12 week blinded treatment phase with placebo crossover followed by 36 weeks of open label treatment phase 16 week treatment trial of placebo vs. adalimumab vs. methotrexate 16 week placebo controlled treatment phase followed by 17 week open-label phase followed by a 19 week blinded placebo controlled withdrawal phase	Subcutaneous: placebo, 80 mg and then 40 mg every other week, 80 mg and then 40 mg weekly; after 12 weeks, placebo group received 80 mg and then 40 mg every other week Adalimumab subcutaneous 80 mg once and then 40 mg every other week; methotrexate orally escalated from 5 mg to 25 mg; Placebo Subcutaneous: placebo, 40 mg every other week	Not reported Not reported 8.8% of adalimumab-treated patients developed antibodies at some point during the study; titers not reported; presence of antibody correlated with loss of response
Ustekinumab	Phase 3 – 766 patients with CPP[14] Phase 3 – 1230 patients with CPP[15]	DB, PC, PG at 48 sites in USA, Canada, and Belgium DB, PC, PG at 70 sites in USA, Canada, Europe	12 week placebo-controlled treatment phase followed by placebo in randomized crossover to treatment group; nonresponders (<50% reduction in PASI) discontinued at week 28 and at week 40 all remaining patients in groups were placed in placebo-controlled randomized withdrawal phase 12 week placebo-controlled treatment phase followed by placebo in randomized crossover to treatment group; nonresponders (<50% reduction in PASI) discontinued at week 28 and at week 28 all remaining patients in groups were placed in randomized dose intensification phase.	Subcutaneous: placebo, 45 mg, 90 mg at week 0 and week 4 and then every 12 weeks; placebo group in randomized crossover to 45 mg or 90 mg at week 12; at week 40 PASI<75 received doses every 8 weeks and all others entered a randomized withdrawal phase Subcutaneous: placebo, 45 mg, 90 mg at week 0 and week 4 and then every 12 weeks; placebo group in randomized crossover to 4 5mg or 90 mg at week 12; at week 28 partial responder received doses every 8 weeks and all others received doses at every 12 weeks	5.1% developed antibodies with titers that were<1:360 At week 52, 12.7% and 2% of partial responders and full responders had antibodies respectively; the antibodies were neutralizing
ABT-874	Phase 2 – 180 patients with CPP[16]	DB, PC, PG at 24 sites in USA and Canada	12 week treatment phase	Subcutaneous: placebo (a), 200 mg once (b), 200 mg weekly for four weeks (c), 100 mg every other week (d), 200 mg every other week (e), 200 mg every week (f)	Not reported

DB = double-blind, PC = placebo controlled, PG = parallel group, CPP = chronic plaque psoriasis, IV = intravenous, PASI = psoriasis area and severity index

**Table 2b:** Efficacies of biologics in clinical trials for psoriasis

Biologic	Efficacy at primary endpoint	Notes
Alefacept	At 2 weeks after treatment phase, reduction in mean PASI (primary end point) was 21%, 38%, 53%, 53% in the placebo, 0.025 mg/kg, 0.075 mg/kg, and 0.15 mg/kg treatment groups, respectively. Patients achieving 75% reduction in PASI were 10%, 21%, 33%, and 31% in the placebo, 0.025 mg/kg, 0.075 mg/kg, and 0.15 mg/kg treatment groups, respectively. At 2 weeks after first treatment phase a 75% reduction in the PASI (primary end point) was 4% and 14% in the placebo and the 7.5 mg treatment groups, respectively. At 2 weeks after treatment phase, reduction in mean PASI (primary end point) was 21%, 34%, 44% in the placebo, 10 mg, and 15 mg treatment groups, respectively	Efficacies of treatment was better than placebo at 12 weeks after treatment phase; higher dropout rate in the placebo group; data collection and analysis was performed by employees at sponsoring company Patients receiving two courses of Alefacept had enhanced control of psoriasis; IV infusion was associated with chills; dose reduced by 33% for subjects that weighed less than 50 kg; no opportunistic infections were noted; higher dropout rate in the placebo group; Study was underpowered at the primary end point of 15% mean reduction of PASI scores at 2 weeks after treatment for 10 mg treatment group; higher dropout rate in the placebo group; data analysis performed by study sponsor
Infliximab	At 10 weeks, a 75% reduction in the PASI (primary end point) was 6%, 72%, 88% in the placebo, 3 mg/kg, and 5mg/kg treatment groups respectively. At 10 weeks, a 75% reduction in the PASI (primary end point) was 3% and 80% in the placebo and infliximab treatment groups, respectively. At 10 weeks, a 75% reduction in the PASI (primary end point) was 1.9%, 70.3%, 75.5% in the placebo, 3 mg/kg, and 5 mg/kg treatment groups, respectively. Higher response efficacies were noted in the scheduled treatment group in comparison to the “as needed” treatment group.	Power analysis not reported; higher dropout rate in the placebo group; site of data analysis not specified; Patient who developed anti-dsDNA did not develop lupus like symptoms; Nail psoriasis improved in treatment group; data analysis performed by study sponsor; Most frequent adverse effects in treatment group were sinusitis and headache; higher dropout rate in the placebo group; site of data not specified; several patients developed lupus like symptoms
Etanercept	At 12 weeks, a 75% reduction in the PASI (primary end point) was 2% and 30% in the placebo and etanercept treatment groups, respectively. At 24 weeks, a 75% reduction in the PASI was 5% and 56% in the placebo and etanercept treatment groups, respectively. At 24 weeks, DLQI improvement was 7% and 65% in the placebo and the 25 mg treatment groups, respectively. At 12 weeks, a 75% reduction in the PASI (primary end point) was 4%, 14%, 34%, and 49% in the placebo, low, medium, and high treatment groups, respectively. At 24 weeks, a 75% reduction in the PASI was 25%, 44%, and 59% in the low, medium, and high treatment groups, respectively. At 24 weeks, DLQI improvement was 7% and 65% in the placebo and the 25 mg treatment groups, respectively. At 12 weeks, a 75% reduction in the PASI (primary end point) was 3%, 34%, and 49% in the placebo, 25 mg, and 50 mg biweekly treatment groups, respectively. At 12 weeks, a 75% reduction in the PASI (primary end point) was 5% and 47% in the placebo and the etanercept treatment groups, respectively. During the open label period, PASI 75 levels decreased with duration of therapy. At 12 weeks, a 75% reduction in the PASI (primary end point) was 11% and 57% in the placebo and the etanercept treatment groups, respectively. Placebo group approached PASI levels of treatment group during open label treatment phase. Withdrawal-retreatment phase data was not reported.	Higher rates of sinusitis and upper respiratory infections in the treatment group; higher dropout rate in the placebo group; site of data analysis not reported; two cases of nonplaque psoriasis reported in treatment group; Not sufficient power to detect difference between placebo and low treatment group; data analysis was performed by the sponsor Retrospective power analysis (?); Higher dropout rate in the placebo group; injection site reactions were worse in the treatment group and were mild to moderate in severity; data analysis performed by sponsor Higher dropout rate in the placebo group; data analysis performed by investigators; tachyphylaxis with duration of therapy although this was related to the presence of antibodies Retrospective power analysis; data storage and analysis performed by the sponsor; higher placebo dropout rate; treatment group had higher rate of streptococcal pharyngitis.
Adalimumab	At 12 weeks, a 75% reduction in the PASI (primary end point) was 4%, 53%, and 80% in the placebo, 40 mg every other week, and 40 mg weekly treatment groups, respectively. Efficacies of achieving PASI 75 decreased with duration of therapy. At 16 weeks, a 75% reduction in the PASI (primary end point) was 18.9%, 35.5%, and 79.6% in the placebo, methotrexate, and adalimumab treatment groups, respectively. At 16 weeks, a 75% reduction in the PASI (primary end point) was 7% and 71% in the placebo and adalimumab treatment groups, respectively. All patients that achieved PASI 75 at week 16 had a 92% improvement in their PASI by week 33. Re-randomization to placebo in withdrawal phase led to higher loss of response.	Efficacy of achieving PASI 75 decreased with duration of therapy; placebo crossover group was similar to treatment group by end of study; site of data analysis not reported Data analysis was performed by sponsor; higher placebo dropout rate; all patients received folate supplementation; methotrexate started low with slow increase of dosage Data analysis performed by sponsor; higher placebo dropout rate; higher injection site reactions in adalimumab treatment group
Ustekinumab	At 12 weeks, a 75% reduction in the PASI (primary end point) was 3.1%, 67.1%, and 66.4% in the placebo, 45 mg, and 90 mg treatment groups, respectively. By week 40, placebo crossover groups had similar efficacies to ustekinumab treatment groups. At 12 weeks, a 75% reduction in the PASI (primary end point) was 3.7%, 66.7%, and 75.7% in the placebo, 45 mg, and 90 mg treatment groups, respectively. Partial responders did not benefit from escalated dosing at 45 mg but had higher PASI 75 rates with escalated dosing at 90 mg.	Data analysis performed by sponsor; higher dropout rate in the placebo group; similar adverse reaction between treatment and placebo groups Data analysis performed by sponsor; higher dropout rate in the placebo group; similar adverse reactions between treatment and placebo group
ABT-874	At 12 weeks, a 75% reduction in the PASI (primary end point) was 3%, 63%, 90%, 93%, 93%, and 90% in the a, b, c, d, e, and f treatment groups, respectively.	Data analysis was performed by sponsor and investigators; high placebo dropout rate; treatment group had higher rate of nasopharyngitis

## Non-cytokine Biologics

### Alefacept

Alefacept was designed to block the CD2/LFA-3 interaction important for T-cell function. Clinical trials with either IV[11,12] or IM[13] alefacept have found it to be effective in the treatment of psoriasis. All of these trials were 12 weeks in length, and tested for improvement at 2 weeks as their primary end point. Efficacy was maintained at 12 weeks. In one of the studies, alefacept was found to reduce the amount of memory effector T-cells without affecting the naïve T-cell population.[11] Another study included three different cohorts with a placebo crossover and placebo withdrawal. This trial found that the cohort that received two courses of alefacept had improved treatment efficiencies compared to the placebo crossover or withdrawal cohorts.[12]

A meta-analysis showed that alefacept treated patients had an overall 9% increased risk of adverse events.[14] The most common adverse effects noted in these studies were dizziness,[11] nausea,[11] infusion-related chills,[11,12] pharygitis,[12,13] headache,[13] and pruritus.[13] A meta-analysis of the safety of alefacept showed coronary artery disease in four subjects, cellulitis in three subjects, and myocardial infarction in three subjects, while none of these serious adverse events were noted in the placebo groups. IV dosing was noted to increase the incidence of serious adverse effects over IM dosing.[14]

Anti-alefacept antibodies were noted to develop in all three studies with up to 4% in the study in which alefacept was administered IM. These antibodies were found to be non-neutralizing.[13] No adverse events were correlated with the presence of the antibody.

### Efalizumab

Efalizumab has been voluntarily withdrawn from the market in the USA partly due to the risks of progressive multifocal leukoencephalopathy. This antibody was manufactured by Genentech and was specific to the CD11a subunit of LFA-1.

## Cytokine Biologics

### Anti-TNF agents

#### Infliximab

Clinical trials with IV infliximab have shown it to have efficacy of reaching a PASI 75 at 10 weeks at 75.5-88% in those treated with 5 mg/kg when compared to 1.9-6% in the placebo group.[15–17] Two of the studies showed that an intermediate dose of 3 mg/kg was also effective in achieving PASI 75 at 10 weeks for 70.3-72% of those treated.[16,17] Efficacies were maintained over placebo for 46-50 weeks, a loss in response was noted in those subjects that developed anti-infliximab antibodies.[15,16] Although not a primary end point, one study noted 26% improvement and a 6% worsening in nail psoriasis of the infliximab and placebo treatment groups, respectively. The most common adverse effects were rhinitis, transaminitis, sinusitis, and headache.[15,16]

#### Etanercept

Phase 2 and phase 3 trials with etanercept delivered subcutaneously report that it is superior to placebo in achieving PASI 75.[18–22] At 12 weeks after treatment, the PASI 75 for biweekly subcutaneous injections of 25 mg or 50 mg were reported at 34% and 49%, respectively, while the placebo group had a 12 week PASI 75 of only 3-4%.[19,21] A dose response was noted from low to high dosing[19,21] and the efficacy continued to increase at 24 weeks.[21] A phase 3 study of a pediatric population revealed that after 12 weeks, subcutaneous dosing at 0.8 mg/kg resulted in 57% of patients receiving a PASI 75 while placebo dosing only achieved a PASI 75 in 11%.[20] Antibody formation against etanercept ranged from 1.1 to 18.3%.[18,19] A loss of response was correlated with the duration of therapy but the formed antibodies were not found to be neutralizing.

The most common side effects noted in adults were upper respiratory tract infections,[22] sinusitis,[22] headaches,[22] and injection site reactions[18,19,21,22] Injection site rections tended to occur more frequently during the first 12 weeks of therapy and approached placebo level frequencies afterward. The most common side effects noted in one pediatric study was an increased incidence of streptoccal pharyngitis and skin papillomas.[20]

#### Adalimumab

Subcutaneously injected adalimumab was found to have superior efficacy of achieving PASI 75 in comparison to placebo in several phase 2 and phase 3 trials. In one phase 2 study, increasing doses of adalimumab were compared against placebo and a dose response was observed. After 12 weeks of therapy it was found that placebo, 40 mg every other week and 40 mg weekly achieved a PASI 75 in 4%, 53%, and 80% of subjects, respectively.[23] Two other phase 3 studies found that 71-79.6% of subjects treated with 40 mg every other week achieved a PASI 75 in comparison to 7-18.9% of those treated with placebo after 16 weeks of treatment.[24,25] The higher rate of efficacy in achieving PASI 75 in the placebo groups of the phase 3 studies may have been related to the 16 week course of treatment in comparison to the 12 week treatment course in the phase 2 study. Antibodies against adalimumab developed in 8.8% of patients at some point during their treatment course and the presence of antibodies was correlated with a loss of response.[25]

One the phase 3 studies compared adalimumab treatment against methotrexate. At 16 weeks, the PASI 75 achieved by subjects in the methotrexate and the adalimumab treatment groups were 35.5% and 79.6%, respectively.[24] Because the methotrexate was started low and increased over time, the 16 weeks observation may have been too short to appropriately assess the methotrexate response.

The most common side effects were upper respiratory infections,[25] nasopharyngitis,[24] headache,[24] and cellulitis.[25]

### Anti-p40 (IL-12/IL-23)

#### Ustekinumab

Ustekinumab is the first of a new class of biological drugs that prevent the actions of IL-12 and IL-23 by binding to their mutual subunit p40. Two phase 3 studies show that subcutaneously injected ustekinumab has superior efficacy in comparison to placebo. Both studies utilized a 12 week placebo controlled period during which ustekinumab had an efficacy of achieving PASI 75 in 66.7-67.1% and 66.4-75.7% in those treated with 45 mg or 90 mg, respectively.[26,27] In the placebo group, 3.1-3.7% achieved PASI 75. Both of these trials included a placebo crossover group that attained similar treatment efficacies as the ustekinumab treatment group. The dosing of ustekinumab is more spaced out than previous biologics with subcutaneous injections given at week 0, week 4, and then at 12 week intervals, making treatment more convenient. The development of antibodies against ustekinumab has been shown to have clinical implications as the antibodies were found to be neutralizing.[26] This study showed that subjects could be split into full responders and partial responders, the latter defined as those subjects that achieved PASI 50 but not PASI 75 by 28 weeks. Partial responders had increased the prevalence of antibodies against ustekinumab.[26]

The most common side effects were injection site reactions.[26] As this is a newly introduced drug, there is little long-term usage studies and post-market surveillance will be important in understanding long-term side effects.

#### ABT-874

ABT-874 is another antibody generated against the p40 subunit and designed to block the actions of IL-12 and IL-23. One phase 2 trial investigated the use of ABT-874 with progressively increasing doses, showing a dose response relationship.[28] While 3% of subjects in the placebo achieved PASI 75 at 12 weeks, 90% of those treated with 200 mg every 4 weeks achieved PASI 75 at 12 weeks. It was found that increasing the dosing beyond 200 mg every 4 weeks did not provide any increase in achieving PASI 75. The most common adverse events were injection site reactions and nasopharyngitis. The development of antibodies against ABT-874 were not reported.

## Discussion

With the growth in development of biological therapies, there are several effective options for the treatment of chronic plaque psoriasis, which is the most prevalent form of psoriasis. Several generalizations can be made from review of the clinical trial literature. It is interesting to note that in most studies, the placebo group had a larger dropout rate than the treatment group [Table 2b], and this may alter the actual differences between the treatment and placebo group. All of the studies compared treatment against placebo, but only one study compared the biological therapy against methotrexate.[24] The formation of antibodies against the biological drug is not uncommon and can affect the long-term efficacy of the biologic. Studies in the use of biological therapies and immunosuppresants for rheumatoid arthritis and Crohn's disease show that combined dosing of a biological agent with another immunosppressive agent, such as methotrexate, decreases the formation of antibodies against the biological agent.[29,30] Although resistance to one biological agent does not imply resistance to another agent, it would be inconvenient to keep switching agents given the chronic nature of psoriasis. A better solution may be to concomitantly treat patients with both a biological agent and another immunospressant, such as methotrexate. Case reports describe the utility in combining methotrexate with a biological agent.[31] However, no studies have investigated the combined therapy of biological agents and methotrexate for psoriasis and currently there are little data on the efficacy or the side effects of combined therapy.

The larger studies reviewed here have focused on the therapy of plaque psoriasis and it is unclear how effective the biological therapies will be in treatment of other forms of psoriasis. Smaller studies have suggested that some of the biologics may be useful for other forms of psoriasis.[32–34]

Unlike the TNF blockers that have been studied for a longer duration of time[35,36] and used extensively in rheumatology, the IL-12/23 blockers are new treatment options and the long-term effects are still largely unknown. Because these biological agents act earlier in the immune response chain, in comparison to the TNF-α blockers, they are potentially more immunosuppressive and thus infection is a concern. In particular, Th-17 cells, whose actions are antagonized by IL-12/23 blockers, are important in protection against bacteria and fungi.[37]

Biologics are generally safe and well tolerated. However, like all medications, they have adverse effects. Importantly, these medications can predispose patients to infections and increased their risk of developing a malignancy.[38–41] All anti-TNF-α agents have been associated with a variety of serious and “routine” opportunistic infections.[38] From a public health standpoint, the development of active tuberculosis in some patients who receive TNF-α inhibitor therapy is a matter of serious concern.[38,39] There is also an increased risk for a variety of malignant conditions such as lymphoma, leukemia, and melanomas.[40,41]

As the use of TNF-α antagonists becomes widespread, further cases of tuberculosis associated with TNF-α blockade can be expected, especially in developing countries with high incidences of tuberculosis.[38] To prevent the reactivation of latent tuberculosis, appropriate screening of patients with Mantoux test and chest X-ray should be performed before initiating anti-TNF therapy, and begin treatment if latent infection is found. The screening strategies employed in Europe and North America have reduced the occurrence of TNF-α inhibitor-associated tuberculosis. Tuberculosis in patients treated with anti-TNF agents may present with extrapulmonary or disseminated disease. Thus, clinicians should be vigilant in monitoring for tuberculosis in their patients treated with TNF-α inhibitors. The role of screening in the prevention of other opportunistic infections is far less certain. No official guidelines currently exist for many of these opportunistic infections, but various authors have made recommendations regarding screening options, as summarized in Table 3.[38,42–44] Because of systemic immune suppression, a variant clinical presentation is expected; atypical signs and symptoms as well as atypical pathogens should be considered. Patients receiving TNF-α inhibitor treatment should be closely monitored for serious infections and should be educated about how to avoid infectious complications.[38] Although rare, clinicians need to closely monitor for malignancy, and induction of autoimmune diseases (psoriasis, lupus) in patients receiving anti-TNF agents.

**Table 3:** Suggested screening tests for certain infections before initiating anti-TNF therapy (39,43,44,45)

Infection	Recommended screening
Tuberculosis	PPD, chest X-ray at baseline and PPD every 12 months.
Histoplasmosis	Consider chest radiograph and urine histoplasmin antigen testing at baseline and every 3 – 4 months for patients who live or have lived in endemic areas.
Coccidioidomycosis	Chest radiograph and serologic testing with IgM and IgG tests at baseline. Consider follow-up testing every 3 – 4 months for patients who live or have lived in endemic areas.

The development of biological therapies has revolutionized psoriasis treatment. Despite the growing number of biological therapies that are entering the clinical arena, many more biological remain on the horizon, including the targeting of IL-21[45] or IL-22.[10] With time, long-term side effects and efficacies will become clearer and help determine which ones are the most suitable for long-term care of psoriasis.
